# Rice SIAH E3 Ligases Interact with RMD Formin and Affect Plant Morphology

**DOI:** 10.1186/s12284-022-00554-8

**Published:** 2022-01-25

**Authors:** Shuwei Chang, Guoqiang Huang, Duoxiang Wang, Wanwan Zhu, Jianxin Shi, Litao Yang, Wanqi Liang, Qi Xie, Dabing Zhang

**Affiliations:** 1 School of Life Sciences and Biotechnology, Shanghai Jiao Tong University, Shanghai, 200240 People’s Republic of China; 2 State Key Laboratory of Plant Genomics, Institute of Genetics and Developmental Biology, The Innovative Academy of Seed Design, Chinese Academy of Sciences, Beijing, 100101 People’s Republic of China

**Keywords:** SIAH, E3 ligase, RMD, Location, Degradation, Height, Seeds, Morphogenesis, Rice

## Abstract

Formins are actin-binding proteins that are key to maintaining the actin cytoskeleton in cells. However, molecular mechanisms controlling the stability of formin proteins in plants remain unknown. Here, we have identified six rice SIAH-type E3 ligases, named RIP1-6 (RMD Interacting Protein 1–6) respectively, with ubiquitination enzyme activity in vitro. All six proteins can form homo- and hetero-dimers with themselves, and hetero-dimers with type II formin RMD/OsFH5. In vivo assays showed that RIP1-6 proteins localize in the cytoplasm with a punctate distribution, and all of them interact with RMD to change its native diffuse cytoplasmic localization to match that of RIP1-6. To our surprise, degradation experiments revealed that RIP1, RIP5, and RIP6 decrease rather than increase the degradation rate of RMD. Genetic analyses revealed redundancy between these six genes; either single or double mutants did not show any obvious phenotypes. However, the sextuple *rip1-6* mutant displayed dwarf height, wrinkled seeds and wider leaves that were similar to the previously reported *rmd* mutant, and defective microfilaments and increased flag leaf angles that were not reported in *rmd* mutant. Collectively, our study provides insights into the mechanisms determining formin protein stability in plants.

**Supplementary Information:**

The online version contains supplementary material available at 10.1186/s12284-022-00554-8.

## Background

E3 ubiquitin ligases, the most abundant eukaryotic proteins (Mazzucotelli et al. 2006; Liyuan et al. 2013; Michael et al. 2015), catalyze the covalent attachment of ubiquitin to substrates with the help of E1 (ubiquitin-activating enzyme) and E2 (ubiquitin-conjugating enzyme) proteins (Buetow and Huang 2016). A wide variety of ubiquitination processes participate in numerous biological pathways (Jmii and Cappadocia 2021; Wu et al. 2021; Liu et al. 2021; Uchida and Kitagawa 2016), and the predominant function, mediated by Lys48-linked ubiquitination, is to target proteins to the proteasome for degradation (Hiranyakorn et al. 2020). Other types of linkages, e.g., Lys63-linkages, signal substrates for non-degradative functions, such as protein assembly or modification (Chen and Sun 2009) (Swatek and Komander 2016); or chromatin modelling or repair (Nakazawa et al. 2020). E3 ligases can also be themselves ubiquitinated, which can affect their activities (Chen et al. 2018).

SINA/SIAH (seven in absentia; seven in absentia homologue) ubiquitin ligases are members of the RING finger E3 ligase family (Qi et al. 2013), and are highly conserved in animals (Pepper et al. 2017) and plants (Den Herder et al. 2008). In these proteins, the N-terminal RING domain binds E2 proteins (Matsuzawa et al. 2003), while the C-terminal SIAH domain contains substrate binding sites and a dimerization domain (Siswanto et al. 2018). SINA E3 ligases engage in a variety of biological processes in plants, such as drought response in rice (Ning et al. 2011), root formation and hormone signaling in Arabidopsis (Xie et al. 2002; Yang et al. 2017), and cold stress in banana (Fan et al. 2017). However, little is known about the role of SINA ligases in cytoskeleton dynamics and microfilament organization.

RMD (also named OsFH5 and BUI1), a type II formin, is involved in actin polymerization. RMD knock out lines show serious morphological defects in vegetable and reproduction stages, such as bent seedlings, stunted adult plants, aberrant inflorescence and seed morphology (Yang et al. 2011; Zhang et al. 2011b), double mutant of RMD and another type II formin OsFH3 showed more severe morphological defects (Chang et al. 2021). *rmd* mutants are also sensitive to gravity response in both above ground and underground parts (Song et al. 2019; Huang et al. 2018). In addition, RMD controls cell growth through auxin–actin regulatory loop (Li et al. 2014). Further elucidation of its interactome and stability regulation will provide novel insights into the formin protein-mediated actin polymerization in plant development.

Here, we report the discovery of six E3 ligases in rice (RIP1 and five homologs) that interact with RMD. All six RIP proteins could form homo- or hetero-dimers, and interacted with RMD in vivo to affect RMD intracellular location and inhibit RMD degradation. Simultaneous mutation of all six *RIP* genes affected young root microfilament formation, and flag leaf and grain morphology, indicating a broad role of RIP proteins at different stages of rice development.

## Results

### RMD Interacts with RIP SIAH Domains In Vivo and In Vitro

**Fig. 1:**
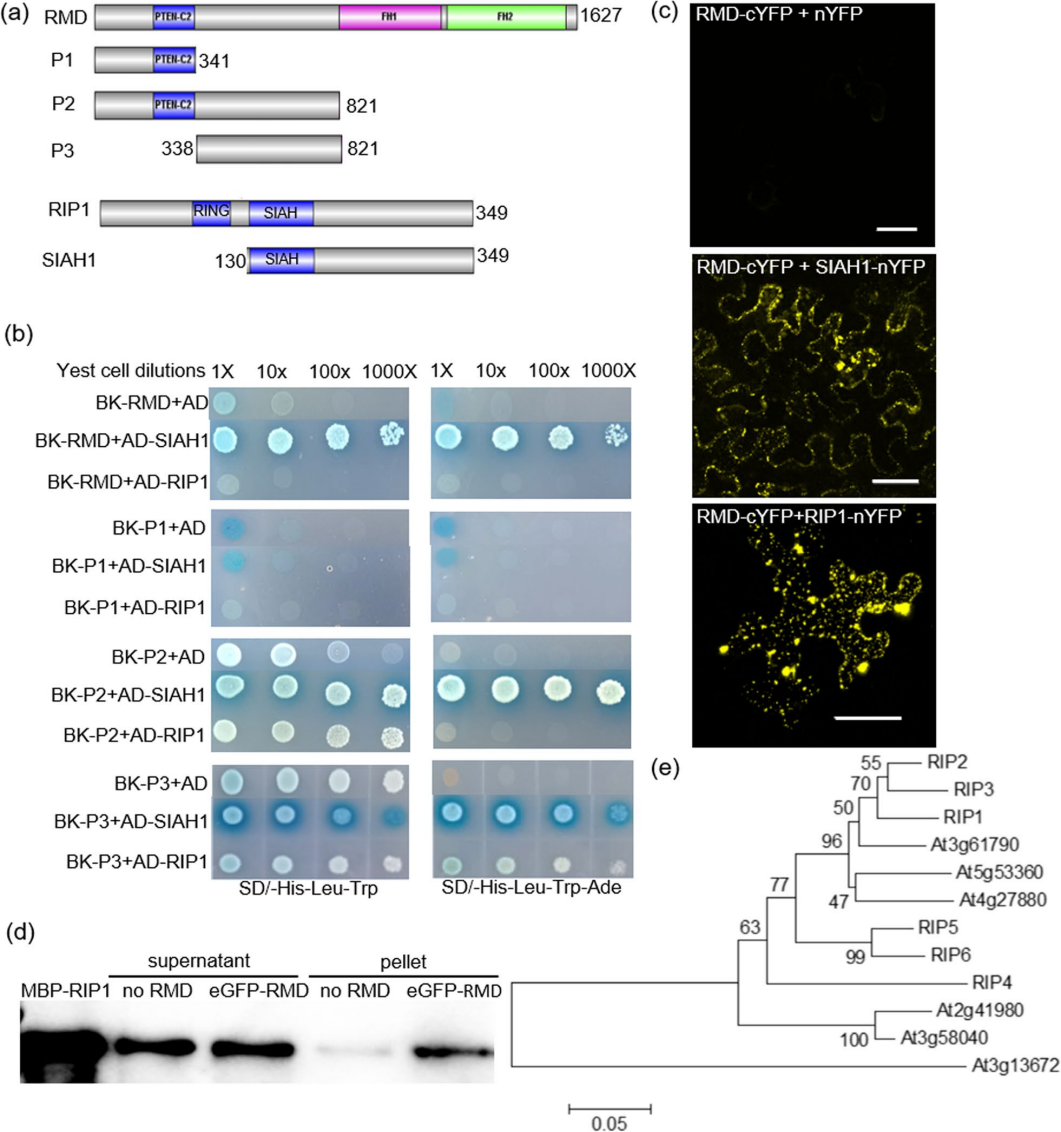
The SIAH domain of RIP1 interacts with RMD. **a** Schematic diagrams of the full-length RMD protein, indicating PTEN-C2, FH1, and FH2 domains; the truncated RMD P1, P2, and P3 constructs; the full-length RIP1 protein, indicating RING and SIAH domains; and the truncated SIAH1 construct. **b** Yeast 2 hybrid (Y2H) experiment revealing that the P3 section of RMD is essential for interaction with the RIP1 SIAH1 domain. Full-length and truncated RMD proteins were expressed from the BK bait vector (pGBKT7), and RIP1/SIAH1 proteins were expressed from the AD prey vector (pGADT7). Yeast cells co-transformed with each bait-prey pair were grown on selective media (-Leu-Trp). **c** Bimolecular fluorescence complementation (BiFC) in tobacco leaves reveals that RMD (fused to cYFP) interacts with the SIAH1 domain of RIP1 (fused to nYFP, middle panel) and full-length RIP1 (fused to nYFP, bottom panel). Bar = 50 µm. **d** Western blot showing that MBP-labelled RIP1 co-immunoprecipitates with eGFP-labelled RMD. RIP1 was purified from *E. coli*, and RMD was extracted from tobacco leaves. RMD was pelleted using anti-GFP immunomagnetic beads, and the presence of RIP1 in the supernatant and pellet, in the presence or absence of RMD, tested using anti-MBP antibody. Lane 1 is a positive control for MBP detection. **e** Phylogenetic analysis of 6 rice RIP and 6 Arabidopsis SIAH proteins. Bootstrap support values are indicated on nodes (%)

RMD contains one N-terminal PTEN (phosphatase and tensin homolog) domain that is involved in the attachment to the plasma membrane, chloroplast, mitochondrion, and/or endoplasmic reticulum (Cvrčková 2013, 2012; van Gisbergen and Bezanilla 2013) and two C-terminal formin homology (FH) domains that are used to bind profilin/actin complex (Zhang et al. 2011b; Yang et al. 2011). To search for proteins that may interact with RMD, we used a yeast two hybrid (Y2H) screen with the RMD N-terminal P2 fragment (821 amino acids) as bait (Fig. 1a). Most of the revealed interacting protein (63%) was the SIAH domain from RIP1 (Fig. 1a). Further analysis with full-length and truncated RMD and RIP1 constructs confirmed that the SIAH domain from RIP1 interacts with the P3 domain of RMD (aa 338–821); this P3 domain has no recognized functional elements or motifs (Fig. 1a, b). Ubiquitous co-expression of *RIP1* and *RMD* genes in rice tissues was confirmed from the RiceFREND database (https://ricefrend.dna.affrc.go.jp/).

Intriguingly, while full-length RMD could interact with the truncated SIAH domain from RIP1, and the full-length RIP1 protein could interact with the truncated P2 and P3 domains from RMD, the two full-length RMD and RIP1 proteins could not interact in the Y2H assay, suggesting possible steric hindrance of two large proteins with the GAL4 activation domain (Fig. 1b). However, these two full-length proteins were observed to interact in bimolecular fluorescence complementation (BiFC) assays in vivo in tobacco leaves (Fig. 1c); and co-immunoprecipitation assays using tagged proteins (RIP1 tagged with maltose binding protein, MBP; RMD tagged with eGFP) also confirmed that the two proteins interact directly in vitro (Fig. 1d).

In silico analyses using the full-length RIP1 protein sequence revealed additional five RIP1 homologs in rice (RIP2-6), and six homologs in *Arabidopsis thaliana*. Phylogenetic analysis revealed similar arrangements in the two species, where RIP1, RIP2, and RIP3 orthologs appeared to form one clade, while the RIP4, RIP5, and RIP6 orthologs formed the other clade (Fig. 1e). Due to the high homology of rice RIP proteins, we tested whether their SIAH domains could interact with RMD, and found that all five SIAH domains (labelled SIAH2-6 from RIP2-6, respectively) can interact with P2 and full-length RMD (Additional file 1: Fig. S1a). Interactions between full-length RIP2, 4, 5, 6 and RMD proteins were verified using BiFC assays in tobacco leaves (Additional file 1: Fig. S1b); however, cloning of the full-length *RIP3* was unsuccessful, possibly due to high GC content, so its expression and interactions could not be analyzed.

### RIP Proteins Can Form Homo- and Hetero-Oligomers

**Fig. 2:**
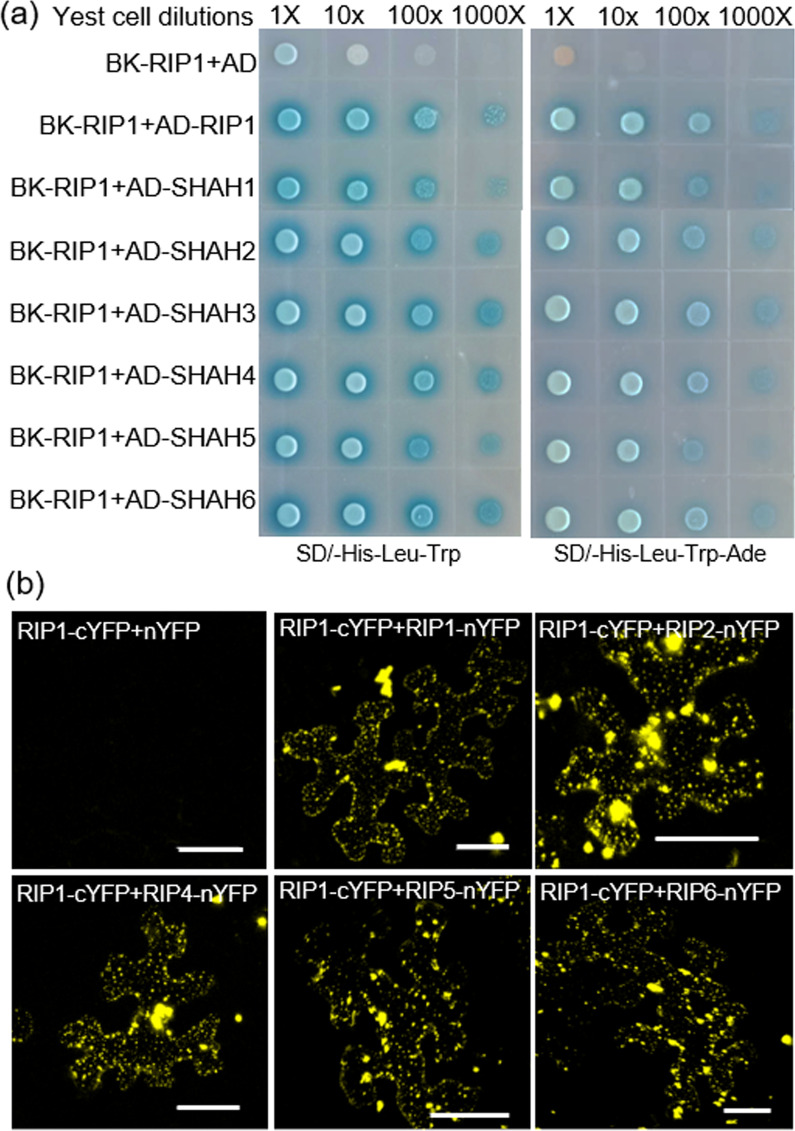
RIP1 forms oligomers with itself and its homologs via SIAH domains. **a** Y2H assays reveal that RIP1 can interact with itself, SIAH1, and SIAH domains from RIP2–RIP6 proteins. **b** BiFC assays confirm that full-length RIP1 (fused to cYFP) interacts with other full-length RIP proteins (fused to nYFP) in tobacco leaves. Bar = 50 µm

Previous reports from Arabidopsis and human research have indicated that E3 ligases can form dimers, mediated by SIAH domains (Xie et al. 2002; Depaux et al. 2006). We used both Y2H and BiFC assays to examine whether this function is conserved in rice RIP proteins. Y2H assays revealed that RIP1 can form homo-oligomers with itself or its SIAH1 domain, and with the SIAH domains of the other five RIP proteins (Fig. 2a). BiFC confirmed that all full-length proteins, when co-expressed either with themselves or with other RIP proteins, interacted in tobacco leaves (Fig. 2b and Additional file 1: Fig. S2). These results suggest possible functional redundancies among rice RIP homologs.

### Interaction with RIP Proteins Affects Localization of RMD

**Fig. 3:**
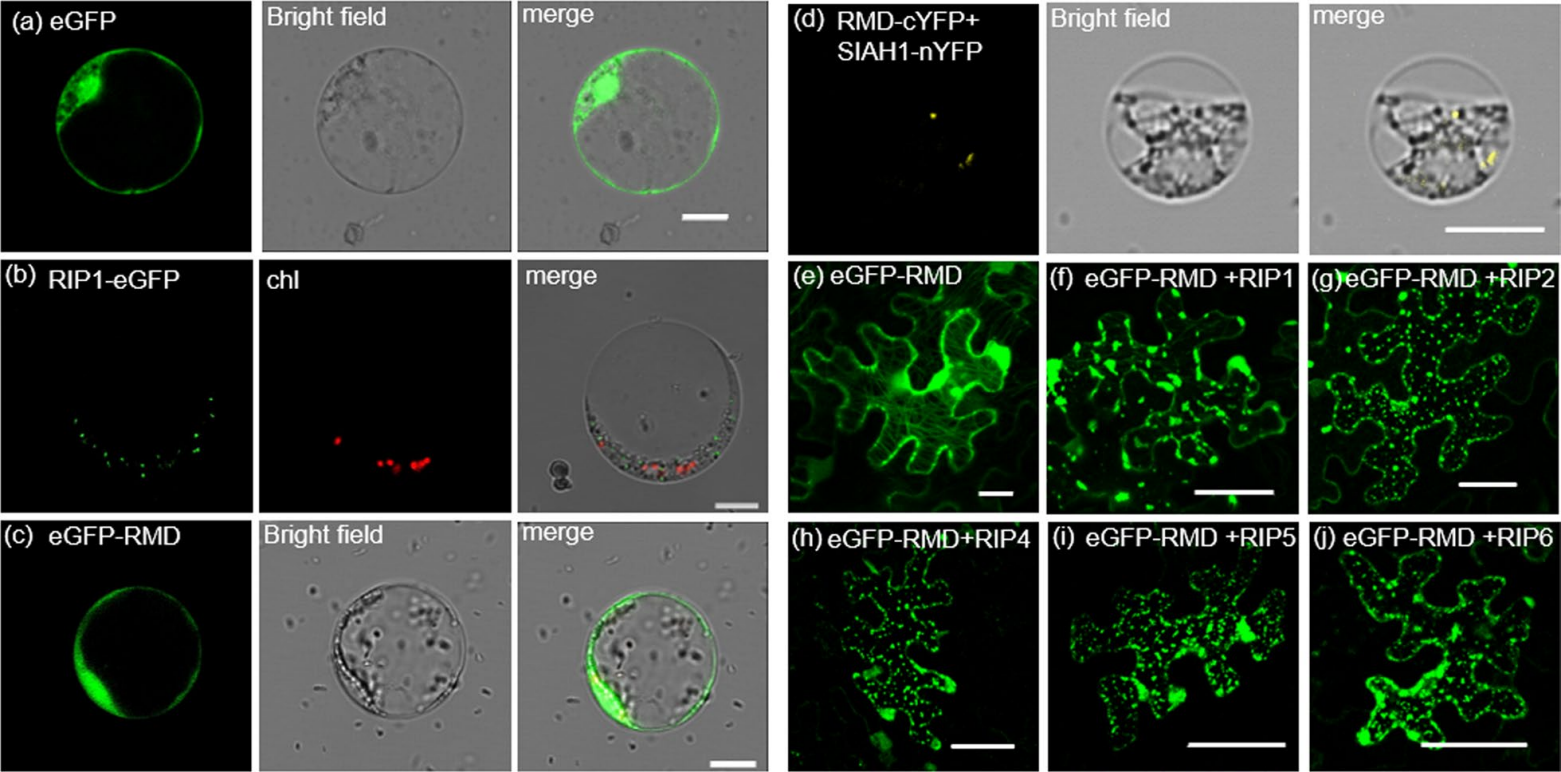
Interaction with RIP proteins influences RMD intracellular localization. **a** RIP1-eGFP, expressed in rice protoplasts, exhibits punctate distribution in the cytoplasm, distinct from chloroplasts (chl) indicated in red via autofluorescence. Bar = 10 µm. **b** eGFP-RMD, expressed in rice protoplasts, occurs diffusely in the cytoplasm and plasma membrane. Bar = 10 µm. **c** BiFC reveals that RMD and SIAH1 interact in the cytoplasm of rice protoplasts and exhibit a punctate distribution. Bar = 10 µm. **d**–**i** eGFP-RMD, expressed in tobacco leaves, occurs diffusely in the cytoplasm and plasma membrane when expressed by itself (**d**); or exhibits punctate distribution in the cytoplasm when co-expressed with RIP1, RIP2, RIP4, RIP5, or RIP6 (**e**–**i**). Bar = 50 µm

To determine the intracellular location of RIP1, we expressed eGFP (as control) and a RIP1-eGFP fusion protein in rice protoplasts. We observed a punctate distribution of RIP1 in the cytoplasm, which is different from eGFP (Fig. 3a, b) but similar to the distribution of the RIP1 homo-oligomer in tobacco leaves (Fig. 2b). We also found that the eGFP-RMD fusion protein is localized at the plasma membrane and the cytoplasm, but in a more diffuse distribution, both in rice protoplasts (Fig. 3c) and tobacco leaves (Fig. 3e). Co-expressing of RMD-cYFP with full length RIP1-nYFP into rice protoplasts was unsuccessful, but co-transfection of RMD-cYFP and the shorter SIAH1-nYFP revealed protein interaction in a punctate pattern in the cytoplasm (Fig. 3d), similar to the pattern observed for full-length RIP1-eGFP (Fig. 3b).

To further explore how RIP proteins affect RMD distribution, we co-expressed eGFP-RMD and RIP proteins into tobacco leaves, and observed the same punctate distribution for all protein combinations (Fig. 3f–j), quite distinct from the pattern observed when eGFP-RMD was expressed alone (Fig. 3e). These results demonstrate that interaction of RMD with RIP proteins appears to alter the intracellular distribution of RMD to align it with RIP proteins.

### RIP Proteins Inhibit Degradation of RMD

**Fig. 4:**
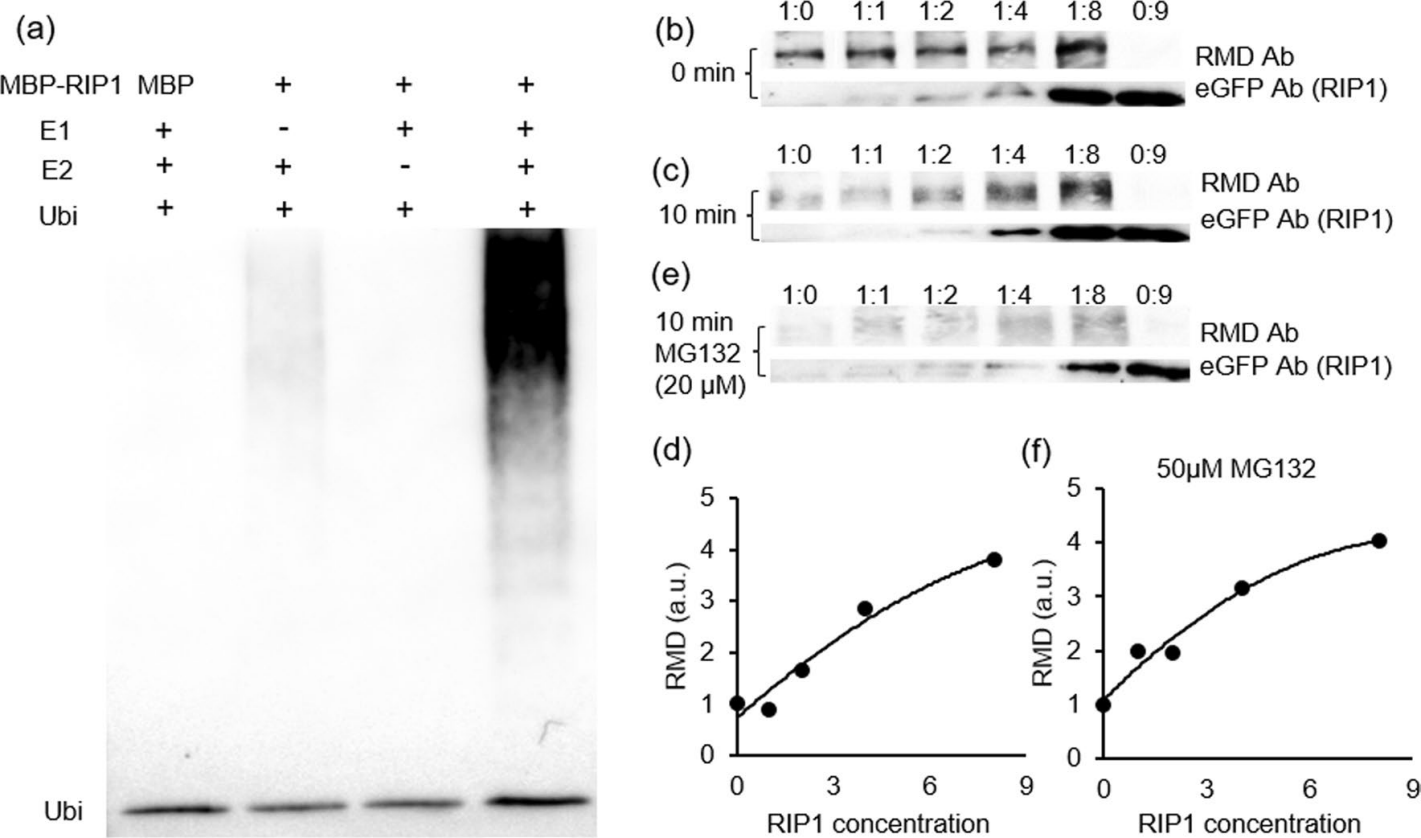
RIP1 ligase has autoubiquitination activity and inhibits degradation of RMD. **a** MBP-labelled RIP1 was assayed for ubiquitination activity in the presence (+) or absence (−) of E1 (from wheat), E2 (AtUBC8) and 6 × his-tagged ubiquitin (Ubi) using anti-ubiquitin antibody. A plain MBP tag was used in the negative control lacking RIP1. **b**, **c**, **e** RMD protein extract was mixed with different ratios (0, 1, 2, 4, or 8 × vol) of RIP1-eGFP extract. The sixth lane contains a 9 × vol of RIP1 extract with no RMD extract. Anti-RMD antibody (Ab) was used to detect RMD; anti-eGFP antibody was used to detect tagged RIP1. Protein amounts: **b** immediately after mixing (0 min); **c** 10 min after mixing; **e** 10 min after mixing in the presence of 50 μM MG132, a proteasome inhibitor. **d**, **f** Quantification of western blot result from (**c**) and (**e**), respectively. a.u., arbitrary unit

The ubiquitination function of RIP1, as a putative E3 ubiquitin ligase, was tested by analyzing autoubiquitination activity in the presence or absence of E1, E2, and ubiquitin. RIP1 only underwent autoubiquitination in the presence of both E1 and E2 (Fig. 4a), confirming its ability to ligate ubiquitin to proteins. This RIP1 function suggests that RIP-RMD interaction may target RMD for ubiquitin-mediated protein degradation via the proteasome system. To test this hypothesis, RMD and RIP-eGFP were separately expressed in tobacco leaves, extracted, and mixed together in different ratios. Presence of RMD or RIP protein was detected by anti-RMD and anti-eGFP antibodies, respectively, at 0 min and 10 min after mixing. In the absence of RIP1, RMD was almost completely degraded after 10 min (Fig. 4b, c). However, RMD degradation was inhibited by the addition of RIP1 (Fig. 4b–d), the higher the RIP1 concentrations, the less the RMD degradation, contrary to expectations that RIP1 would increase RMD degradation. Addition of the proteasome inhibitor MG132 didn’t affect the pattern of RMD degradation (Fig. 4e, f), suggesting that RIP1 does not function in targeting RMD for proteasome-mediated degradation. Further experiments with RIP5 and RIP6 showed similar results, indicating functional conservation among RIP proteins (Additional file 1: Fig. S3).

### The Sextuple *rip1-6* Mutants show Developmental Defects Similar to *rmd*

**Fig. 5:**
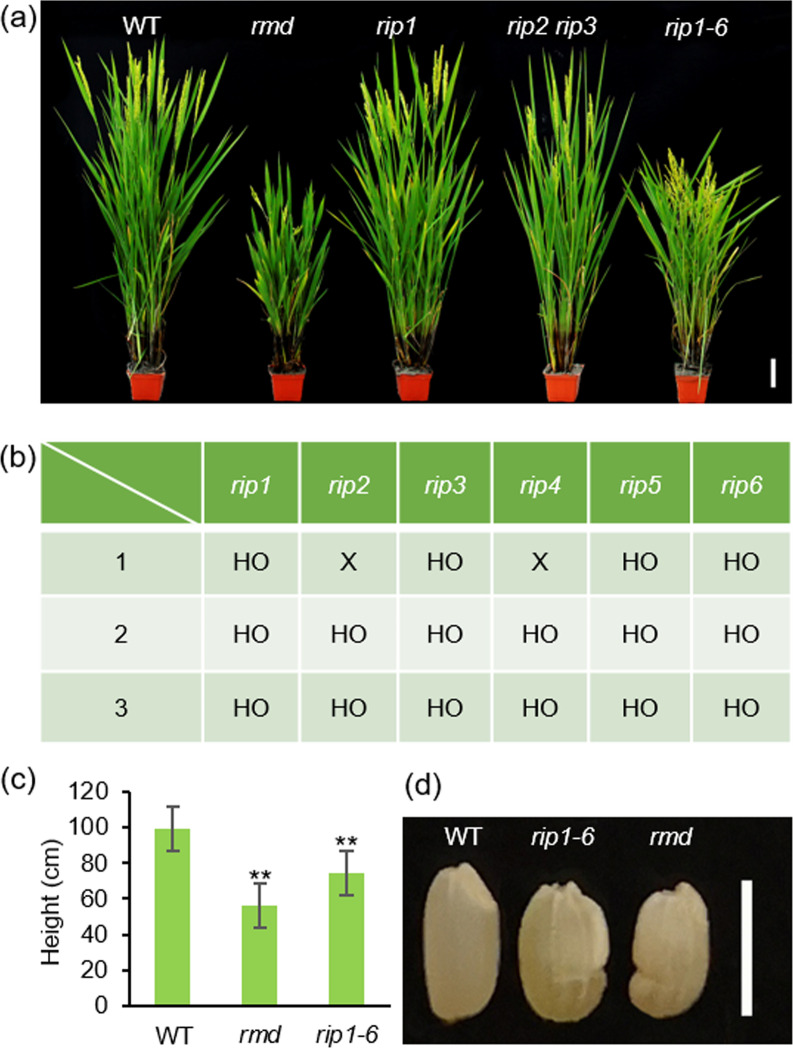
Construction and phenotypes of *rip* single, double, and sextuple mutant lines. **a** Phenotype of *rip1*, *rip2 rip3*, and *rip1–6* plants compared with wild type (WT) 9522 and *rmd* plants at heading stage. Bar = 10 cm. **b** Three *rip1-6* mutants were generated. HO, homozygote; X, amino acid(s) deletion but no change in frame/premature truncation. All HO mutations resulted in premature protein truncation (see Additional file 1: Fig. S5). **c** Height of mature WT, rmd, and rip1–6 plants. n = 15, **P < 0.01 (Student’s t-test). **d** Phenotype of WT, rmd, and rip1–6 seeds, showing defects in seed length and smoothness. Bar = 0.5 cm

To study the functions of RIP proteins in vivo, we generated loss-of-function and overexpression mutants of individual *RIP* genes and found that mutation or overexpression of the single *RIP1* gene did not show any obvious phenotype (Fig. 5a and Additional file 1: Fig. S4). We also generated *rip2 rip3* double mutant, however, it did not show any obvious phenotype different from WT (Fig. 5a).

Nevertheless, we observed significant developmental defects in three sextuple mutant lines that simultaneously knocking outing of all 6 *RIP* genes (*rip1-*6; Fig. 5a, b and Additional file 1: Fig. S5). All three *rip1-6* mutants exhibited the same phenotypes that are similar to *rmd* mutant. Compared with WT, *rip1-6* mutants had shorter height (Fig. 5c) and smaller grains with misshaped surface (Fig. 5d and Additional file 1: Fig. S6a–c), However, *rip1-6* plants also exhibited larger flag leaf angle (Additional file 1: Fig. S6d and S6e) and increased width of flag leaves (Additional file 1: Fig. S6f and S6g) that was not observed in *rmd* plants.

### Microfilaments in *rip1-6* Roots Become Tangled

**Fig. 6:**
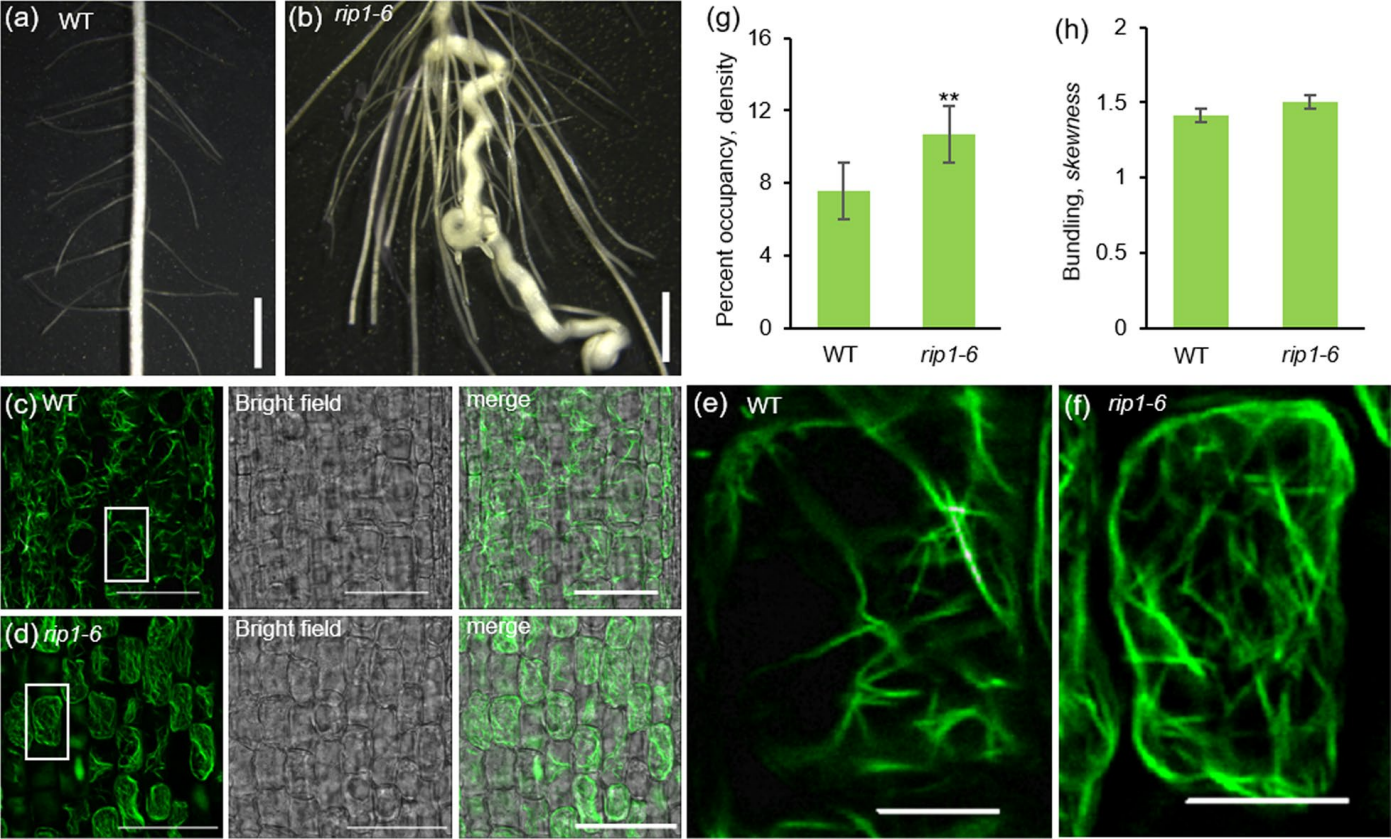
Mutant *rip1–6* lines exhibit microfilament defects. **a**, **b** Roots of 5 days old wild type (WT) and *rip1–6* plants. Bar = 2 mm. **c**, **d** WT and *rip1–6* root tip microfilaments stained with phalloidin. Bar = 50 µm. **e**, **f** Enlargements of boxed areas in (**c**) and (**d**), respectively. Bar = 10 µm. **g** Actin filament abundance, or percentage of occupancy, in WT and *rip1–6* root tips. n = 40, **P < 0.005 (Student’s t-test). **h** Actin filament bundling, or skewness, in WT and *rip1–6* root tips. n = 40

Compared with WT, *rip1-6* roots displayed a spiral and crinkled growth (Fig. 6a, b), which was different from *rmd* mutant roots that showed a wavy and curve growth (Yang et al. 2011). Given the interaction between RIP proteins and RMD, and the role of RMD in microfilament extending and bundling, we examined the morphology of root microfilaments using phalloidin-488 staining and found that microfilaments in *rip1-6* roots were relatively tangled compared with WT (Fig. 6c–f). In addition, microfilaments in *rip1-6* roots were concentrated at cell boundaries with an obvious increase in actin filament abundance but without measurable change in microfilament bundling (Fig. 6g, h).

## Discussion

E3 ligases play important roles in multiple biological pathways in plants (Liu et al. 2021; Uchida and Kitagawa 2016), via targeting proteins to the proteasome for degradation (Swatek and Komander 2016), or through other mechanisms including protein assembly or modification (Chen and Sun 2009; Swatek and Komander 2016). Here, we report the discovery of six redundant RIP proteins that can interact with RMD via their SIAH domains, and the redundant function of them in rice development through interacting with a known formin protein, RMD.

RIPs contained the SIAH domain and it was the P3 fragment in the SIAH domain that interacted with RMD. A short peptide motif (PxAxVxP; VxP, core sequence; x is any amino acid) has been reported in substrates of other SIAH ubiquitin ligases to mediate interaction with SIAH ligases (House et al. 2003). A PKAVKP motif found at the C-terminal end of the P3 fragment of RMD (Fig. 1a) explained why P3 and P2 but not P1 fragments can interact with SIAH domain of RIP1 (Fig. 1b). Our results also showed that the PTEN-C2 domain in P1 and P2 has no role in RIP-RMD interaction, therefore, the un-identified specific domains or elements within the P3 fragment of RMD mediates protein interactions between RIPs and RMD. Consistent with other SINA ligases, such as MtSINA in *Medicago truncatula* (Den Herder et al. 2008), SlSINA in tomato (Wang et al. 2018), and MdSINA2 in apple (Li et al. 2020), these identified RIP proteins in rice could form homo- or hetero-oligomers (Fig. 2 and Additional file 1: Fig. S2).

Although most SIAH-type E3 ligases are located at the nucleus (Table 1), other SIAH-type E3 ligases are also located to the cytoplasm (Yang et al. 2017), endosomal and autophagic vesicles (Xia et al. 2020), or the plasma membrane (Lim et al. 2014). In this study, we found that RIP1 is localized to specific points in the cytoplasm distinct from chloroplasts (Figs. 2b, 3b), and that RIP1 can influence localization of RMD protein. RMD localized diffusely to the cytoplasm and the plasma membrane in the absence of RIP1-6 proteins (Fig. 3c, e) while RMD adopted a punctate distribution in the cytoplasm similar to those of RIPs in the presence of RIP1-6 proteins (Fig. 3d, f–j). This result was consistent with several reported studies in other plant species, where SINA4 relocates SYMRK in *Lotus japonicus* (Den Herder et al. 2012), and SINAT5 relocates FLC in Arabidopsis (Park et al. 2007). Our results also confirmed the research in yeast, in which Ring type E3 ligases (Dma1 and Dma2) interact with formin Bni1 and Bnr1, and are required for their proper localization (Juanes and Piatti 2016). As RMD is associated with amyloplasts in the shoot (Song et al. 2019), and RMD shows a punctate distribution (Fig. 3d, f–j) in the presence of RIPs, it is interesting to investigate if RIPs control amyloplast localization of RMD in roots.

**Table 1:** SIAH E3 ligases identified in plants

Gene	Species	Location	Substrate	Function	References
*OsDIS1*	Rice	Nucleus	OsNek6	Degradation	Ning et al. (2011)
*OsHIR*_*1*_	Rice	Plasma membrane, nucleus	OsTIP4;1	Degradation	Lim et al. (2014)
*SINAT E3*	Arabidopsis	Punctate structures in the cytoplasm, nucleus	FREE1, VPS23A	Degradation	Xia et al. (2020), Xiao et al. (2020)
*MaSINA1*	Banana	Nucleus	MaICE1	Degradation	Fan et al. (2017)
*SINAT5*	Arabidopsis	Nuclear body	LHY, DET1	LHY is a substrate of SINAT5, DET1 inhibits E3 ligase activity of SINAT5	Park et al. (2007, 2010)
*MdSINA2*	Apple	Nucleus	**–**	–	Li et al. (2020)
*SINA4*	*Lotus japonicus*	Punctate at the cytosolic side of plasma membrane	SYMRK	Affects SYMRK stability	Den Herder et al. (2012)

– not reported

We found that interactions between RIPs and RMD prevent but not promote the degradation of RMD, which was not in agreement with most of reported SIAH ubiquitin ligases that mediate degradation of target proteins (Table 1). A previous study in Arabidopsis showed that a SIAH ubiquitin ligase, SINAT5, has dual functions. SINAT5 has two substrates: LHY and DET1. While LHY can be ubiquitinated by SINAT5 for degradation, DET1 cannot be ubiquitinated by SINAT5 but the presence of SINAT5 indirectly inhibits LHY proteolytic turnover (Park et al. 2010). Although the formin mDia2 was reported to be degraded through ubiquitin, which E3 is involved in this process remains unknown (DeWard and Alberts 2009). Therefore, our study has described how a SIAH ligase, RIP1, directly interacts with a formin protein (RMD) to inhibit its degradation (Figs. 1d, 4a, d and Additional file 1: Fig. S3). It is not known whether RMD is a direct ubiquitination substrate of RIP1. We have shown that RIP1 has autoubiquitination activity (Fig. 4a), but in vitro analysis of RMD ubiquitination was not successful due mainly to the failure in the extraction of RMD; its high molecular weight (~ 180 kDa) and high proline content that hindered extraction and purification. Nevertheless, we have also shown clearly that RMD degradation was inhibited by RIP1 in a nearly RIP1 concentration-dependent manner (Fig. 4d), which was not affected by MG132 treatment (Fig. 4e, f). Our study, thus, provides evidence for the establishment of the relationship between SIAH type E3 ligases and formin protein turnover in rice.

SIAH type E3 ligases are evolutionarily conserved, often presenting as multiple copies with high homology (Hu et al. 2021; Yang et al. 2017). RIP2-6, just like RIP1, could all interact with RMD and affect RMD intracellular location. Neither *rip1* nor *rip2 rip3* mutants showed any obvious phenotype while *rip1-6* showed significant defects, suggesting functional redundancy among the RIP1-6 homologs. *rip1-6* mutant showed similar phenotypes to that of *rmd*, providing supportive evidence for our findings that RIP1-6 do not promote RMD degradation.

Further evidence for the function of RIP1-RIP6 in modifying the actin cytoskeleton came from analysis of root microfilaments in plants lacking all 6 *RIP* genes (Fig. 6). Roots in young *rip1-6* seedlings exhibited a highly twisted phenotype (Fig. 6a, b), likely caused by an increase in microfilament density and alterations in microfilament distribution within the root cells as observed in this study (Fig. 6c–g). As RIP proteins stabilizes RMD, compared with WT, *rip1-6* mutant may lack enough RMD to maintain roots’ normal gravity response (Huang et al. 2018), which results in twisted growth. The weak twisted root phenotype was also observed *rmd* plants with perturbations in microfilament organization (Zhang et al. 2011b). However, compared with *rmd* mutant, the increase of microfilament density in *rip1-6* is obvious (Fig. 6e, f) (Zhang et al. 2011b), we speculate that this change, likely derived from the effect of RIP on other proteins associated with microfilament organization, may hinder information exchange and material transport between cells, leading to abnormal root growth. RIP We also stained coleoptiles (3–5 mm in length) of WT and *rip1-6* with phalloidin-488 but didn’t observed any obviously differences in microfilament density and alterations in microfilament distribution between *rip1-6* and WT (Additional file 1: Fig. S7). One possibility could be that *rip1-6* coleoptiles looked similar to WT; the second reason could be derived from tissue-specific effect of RIP1-RIP6. Nevertheless, currently, we cannot exclude the possibility that observed changes in microfilament organization in *rip1-6* roots is not directly related to F-actin polymerization but a secondary effect of cell differentiation and cell morphology deformation, further research will be required to unravel the molecular mechanisms involved.

Similar to *rmd* plants, *rip1-6* plants were shorter with shorter, crinkled grains and wider flag leaves (Fig. 5 and Additional file 1: Fig. S6). Different from *rmd* plants, the angle between the flag leaf and the stem was increased in *rip1-6* plants (Additional file 1: Fig. S6d and S6e). While the mechanism or biological role of this change is not known, this result indicates that RIP1 participates in several interacting regulatory networks, similar to other E3 ligases, e.g., SDIR1 that targets SDIR1-INTERACTING PROTEIN1 for degradation but also modulates the salt stress response and ABA signaling in Arabidopsis (Zhang et al. 2015).

## Conclusions

This work has identified a SIAH protein, RIP1, and its homologous proteins (RIP2-RIP6). All RIPs can form homo- and hetero- oligomers in vivo and all can interact with the RMD formin protein. These six RIPs function redundantly affecting plant growth at multiple stages of rice development, likely through redirecting the intracellular location of RMD and inhibiting RMD degradation.

## Materials and Methods

### Plant Materials and Growth Conditions

WT rice (*Oryza sativa* cv. 9522) and mutant plants were grown in the paddy fields of Shanghai Jiao Tong University (30° N 121° E) from June to September (the natural growing season) according to standard local practice. Stem lengths were measured, and grains were harvested, at maturity. Differences in plant height and flag leaf angle at heading stage, leaf width at young seedling stage, and seed length/width at grain maturity were measured using a ruler. The *rmd* mutant in the 9522 background was available from previous work (Zhang et al. 2011a, b). The single *rip1*, double *rip2 rip3*, and sextuple *rip1-6* mutants were generated in 9522 via CRISPR-Cas9 as previously described (Xie et al. 2015) using CRISPR-Cas9 guide RNAs and screening primers shown in Additional file 2: Table S1. *RIP1* overexpression lines were generated by cloning the *RIP1* coding sequence into pTCK303 under control of the ubiquitin promoter (primers in Additional file 2: Table S1), and transforming this construct into 9522 calli, as previously described (Bevitori et al. 2014).

### Yeast Two Hybrid (Y2H) Assays

Yeast two hybrid experiments were performed according to the manufacturer’s instructions (Clontech). To assess interactions between RMD and RIP1, the coding sequences of *RMD*, *P1*, *P2* and *P3* were fused in-frame with the sequence encoding the GAL4 DNA-binding domain of the bait vector pGBKT7 (BK), while the coding sequences of *RIP1* and *SIAH1* were cloned in the prey vector pGADT7 (AD). To analyze formation of homo- and hetero-oligomers between RIP proteins, the *RIP1* coding sequence was cloned into pGBKT7, and paired with prey vectors encoding SIAH1–6 domains from RIP1 to RIP6, respectively. Primers for cloning are shown in Additional file 2: Table S1.

Each bait–prey pair was co-transformed into *Saccharomyces cerevisiae* strain AH109 as previously described (Lecrenier et al. 1998). Yeast cells were grown for 2–3 days in liquid YPDA (yeast extract peptone dextrose adenine) medium at 28 °C, then dilutions were spotted onto selective media (–his –leu –trp or –his –leu –trp –ade) containing 20 mg/ml X-gal, and grown for a further 2–3 days.

### Bimolecular Fluorescence Complementation (BiFC) and Tobacco Transformation

cDNAs encoding *RIP1*-*RIP6*, *SIAH1*, and *RMD* were amplified and fused with nYFP, cYFP, and/or eGFP, cloned into pXY104 and pXY106 vectors (kindly provided by Prof. Hongquan Yang, SJTU), using primers shown in Additional file 2: Table S1. Constructs were individually or co-transformed into 2-day *Nicotiana benthamiana* leaves using *Agrobacterium*-mediated transformation as previously described (Li et al. 2017). After 24–36 h in the dark, tobacco leaf cells were observed under 50% glycerol with a Leica sp5 confocal laser scanning microscope. eGFP excitation wavelength was 488 nm, emission wavelength was 470–600 nm; YFP excitation wavelength was 514 nm, emission wavelength was 485–625 nm.

### Co-Immunoprecipitation Experiments and Western Blotting

RIP1 tagged with maltose binding protein (MBP) was generated and expressed in *E. coli* and purified according to the pET manual. eGFP and eGFP-RMD were constructed into pCAMBIA1301 and pXY104 vectors, separately expressed in tobacco, proteins were extracted with buffer (20 mM Hepes–KOH at pH 7.5, 40 mM KCl, 1 mM EDTA, 0.5% (vol/vol) Triton X-100, and PMSF) obtained from New England Biolabs (Geldner et al. 2001), magnetic beads GFP-Trap® (ChromoTek) conjugated with GFP antibodies pre-washed then adding into eGFP and eGFP-RMD extracting solution, and also purified MBP-RIP1, incubate at 4 °C for 4 h and rotated slowly. Centrifuge at 14,000 g 4 °C for 15 min, transfer the supernatant to new tubes. 5 × protein loading buffer was added separately to supernatant and sediment samples, boiled for 5 min, and 8 µl boiled sample was used for western blotting, anti-MBP antibody (Abcam) was used to detected RIP1.

### In Silico Analyses

The RIP1 protein sequence was used for BLASTp screening of other proteins from National Center for Biotechnology Information (NCBI) and https://www.arabidopsis.org/. Matching protein sequences were aligned with the software Molecular Evolutionary Genetics Analysis (MEGA) and used to create an evolutionary tree. Branch support was assessed with 1000 bootstrap replicates (Felsenstein 1985; Sanderson and Wojciechowski 2000).

https://ricefrend.dna.affrc.go.jp/ was used to analyze gene expression in different organs and different periods in rice.

### Transient Gene Expression in Rice Protoplasts

Rice protoplasts were prepared according to Zhang et al. (2011a) with the following modifications. WT 9522 seeds were germinated in the dark for 2 weeks. 50–60 seedlings (no seeds) were harvested, cut into 0.5 mm lengths, and incubated in 0.6 M mannitol in the dark for 10 min. The mannitol solution was discarded and replaced with enzymatic solution (1.5% (w/w) cellulase RS, 0.75% (w/w) macerozyme R-10, 0.6 M mannitol, 10 mM MES (pH 5.7), 10 mM CaCl_2_, and 0.1% BSA), and vacuum infiltrated for 1 h, before shaking at 60–80 rpm for 4–5 h in the dark at room temperature. The enzymatic hydrolysate was removed by aspiration. Cells were gently resuspended in W5 solution (154 mM NaCl, 125 mM CaCl_2_, 5 mM KCl, and 2 mM MES (pH 5.7)), and the protoplasts were released after shaking for 1 h in the dark at room temperature. Protoplasts were collected after filtration through 40 μm nylon mesh (Millipore) and washed 3 times with fresh W5 solution, pelleting at 200 g for 3 min between washes. Protoplasts were washed a final time in MMG solution (0.4 M mannitol, 15 mM MgCl_2_ and 4 mM MES (pH 5.7)), and resuspended to a concentration of 2 × 10^6^ cells/ml.

For transient protoplast transformation, 10 μg of plasmid DNA and 200 μl of protoplast solution were combined in a 2 ml Eppendorf tubes, to which 220 µl (w/v) PEG_4000_ was gentled added, and mixed gently at room temperature for 20 min. 1 ml of W5 solution was added, and protoplasts gently pelleted at 200 g for 1 min. The supernatant was discarded, and the pellet resuspended in 1 ml W5 solution. Protoplasts were transferred to a 12-well plate (moistened in advance with 1 mL 5% BSA than removed). Plates were incubated in the dark at 22 °C overnight, and again pelleted gently at 200 g for 1 min. The supernatant was discarded, and the fluorescence signal was observed by confocal fluorescence microscopy. All protoplast transformation experiments were repeated fat least three times. Excitation wavelength for eGFP was 488 nm; for YFP was 514 nm; while chloroplasts fluoresced spontaneously.

### Autoubiquitination Assay

Reactions were performed in 30 µl volume, including 3 µl 10 × reaction buffer (0.5 M Tris–HCl, 100 mM MgCl_2_, 50 mM ATP, 20 mM dithiothreitol), 50 ng E1, 200–500 ng E2, 500 ng E3, and 5 µg ubiquitin. Control reactions minus E1 and E2 were performed at the same time. Reactions were incubated at 30 °C for 1.5 h with agitation (900 rpm) in a thermomixer, and stopped by adding 7 µl 5 × protein loading buffer and boiling for 5 min. 5 µl boiled sample was used for western blotting, anti-ubiquitin antibody (Sigma) was used to detected RIP1 ubiquitination, as described above.

### In Vitro Protein Stability Assays

Vectors expressing RMD, RIP1-eGFP, RIP5-eGFP, and RIP6-eGFP (as described above) were introduced separately into *N. benthamiana* leaves via *Agrobacterium*-mediated transformation, as described above. After 36–48 h in the dark, tobacco leaves were harvested, ground under liquid nitrogen, and extracted with a non-denaturing extraction buffer (50 mM Tris-MES (pH 8.0), 0.5 M sucrose, 1 mM MgCl_2_, 10 mM EDTA, 5 mM DTT, and protease inhibitor cocktail (Sigma, 1 complete Mini tablet per 10 ml of extraction buffer)).

RMD extract was mixed with different ratios (0, 1, 2, 4, or 8 × vol) of RIP-eGFP extract, and incubated at room temperature for 0 min, or for 10 min in the presence or absence of 20 μM MG132 (Sigma), a proteosome inhibitor. A negative control lacking RMD (a 9 × vol of RIP1 extract) was also used. Mixtures were run on a protein gel, transferred to a western blot, and probed with anti-RMD antibody (Zhang et al. 2011b) or anti-eGFP antibody to detect RIP protein, as previously described. Experiments were repeated three times. Band quantification was performed using image J software.

### qRT-PCR Assays

Total RNA was isolated from WT and *RIP1* overexpression transgenic lines using Trizol reagent (Generay) according to manufacturer’s instructions. After treatment with DNase (Promega), 0.3 mg RNA was used to synthesize the oligo(dT) primed first-strand cDNA using the ReverTra Ace-a-First Strand cDNA synthesis kit (TOYOBO) according to manufacturer’s instructions. qRT-PCR analysis was performed using SYBR Premix EX Taq (TaKaRa) on a Rotor-Gene RG3000A detection system (Corbett Research) with the exception of the annealing temperature at 55 °C when using the primers RTRIP1S and RTRIP1A (Additional file 2: Table S1). Results were normalized to expression of 18S RNA. RT-PCR analysis was performed as qRT-PCR without SYBR Green I. Three biological replicates were used, and each with three technical repeats.

### Root and Microfilament Observation

Roots from 3 d seedlings grown in light incubator in water were photographed with a Leica camera.

Microfilaments from cv. 9522 and *rip1-6* roots were stained using the glycerol method (Olyslaegers and Verbelen 1998). Roots from 3 d seedlings (grown as above) were incubated in PEM buffer (100 mM PIPES, 10 mM EGTA, 5 mM MgSO_4_, and 0.3 M mannitol, pH 6.9) that contains 1% (w/v) glycerol (Sigma Aldrich) and 6.6 mM Alexa Fluor 488-phalloidin staining (Invitrogen). After a 30 min incubation, root tips were observed in 50% glycerol with a Leica TCS SP5 confocal laser scanning microscope equipped with a 363 1.46-numerical aperture HC PLANs objective to determine microfilament lengths in lateral root cells. Actin filament abundance and bundling (*skewness*) were measured using image J.

### Accession Numbers

*RIP1*, Os02g0293400; *RIP2*, Os05g0238200; *RIP3*, Os01g0234900; *RIP4*, Os02g0128800; *RIP5*, Os03g0356414; *RIP6*, Os07g0659800. Arabidopsis *RIP* homologs: At2g41980, At3g13672, At3g58040, At5g53360, At4g27880, At3g61790.

## Supplementary Information

**Additional file 1: Fig. S1.** RIP1 has five rice homologs that all interact with RMD. **Fig. S2.** RIP proteins form homo- and hetero-dimers. **Fig. S3.** RIP5 and RIP6 inhibit degradation of RMD. **Fig. S4.** *RIP1* overexpression lines have no obvious phenotype. **Fig. S5.** Changes in coding sequence (CDS) and protein amino acid (aa) sequence for 3 independent mutants for the 6 rip genes. **Fig. S6.** Other rip1-6 phenotypes.

**Additional file 2: Table S1.** Primers used in this study.

## Data Availability

All data generated or analyzed during this study are included in this published article and its supplementary information files.

## Abbreviations

Ab: Antibody
ade: Adenine
BiFC: Bimolecular fluorescence complementation
BUI1: Bent uppermost internode1
cDNA: Complementary DNA
Chr: Chromosome
CRISPR-Cas9: Clustered regularly interspaced short palindromic repeats–CRISPR-associated protein 9
cYFP: C-terminal domain of yellow fluorescent protein
eGFP: Enhanced green fluorescent protein
FH: Formin homology domain
HO: Homozygous mutant
MBP: Maltose binding protein
nYFP: N-terminal domain of yellow fluorescent protein
PTEN: Phosphatase and tensin homolog
RIP: RMD interacting protein
RMD: Rice morphology determinant
SIAH: Seven in absentia homologue
SINA: Seven in absentia
Y2H: Yeast two hybrid
