# Supplementary material for: Rice SIAH E3 Ligases Interact with RMD Formin and Affect Plant Morphology
Source: Rice (N Y). 2022 Jan 25;15:6. doi: 10.1186/s12284-022-00554-8 (PMC8786996; doi:10.1186/s12284-022-00554-8)
Supplement: Supplementary file 1 — Additional file 1: Fig. S1. RIP1 has five rice homologs that all interact with RMD. Fig. S2. RIP proteins form homo- and hetero-dimers. Fig. S3. RIP5 and RIP6 inhibit degradation of RMD. Fig. S4. RIP1 overexpression lines have no obvious phenotype. Fig. S5. Changes in coding sequence (CDS) and protein amino acid (aa) sequence for 3 independent mutants for the 6 rip genes. Fig. S6. Other rip1-6 phenotypes. [file 12284_2022_554_MOESM1_ESM.docx]

**Additional file 1: Supplemental Figures**


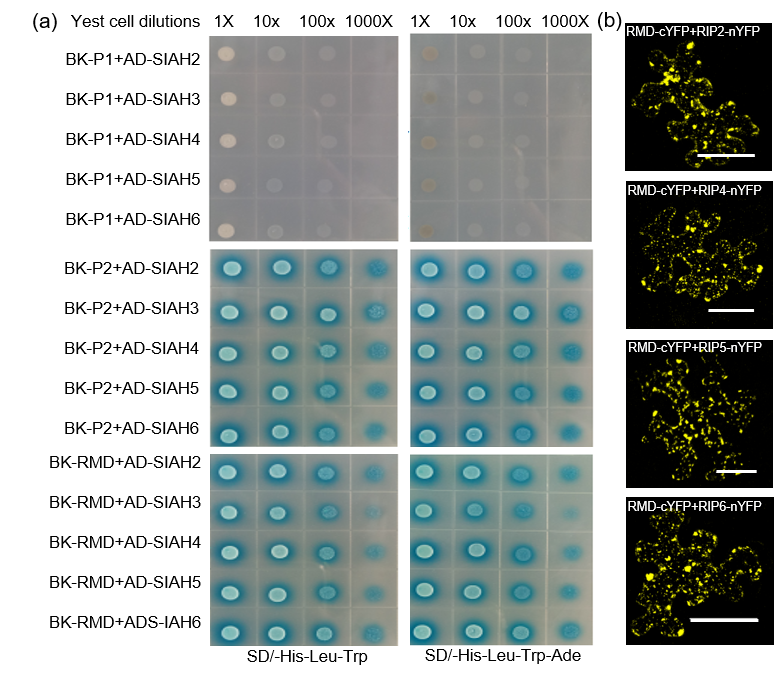


**Figure S1.** RIP1 has five rice homologs that all interact with RMD.

(a) Yeast 2 hybrid experiment, revealing that the P2 section of RMD is essential for interaction with SIAH domains of all RIP proteins. Full-length and truncated RMD proteins were expressed from the BK bait vector (pGBKT7), and SIAH domains from RIP2–RIP6 proteins were expressed from the AD prey vector (pGADT7). Yeast cells co-transformed with each bait–prey pair were grown on selective medium (-His-Leu-Trp and -His-Leu-Trp-Ade).

(b) Bimolecular fluorescence complementation in tobacco leaves reveals that RMD (fused to cYFP) interacts with RIP2, RIP4, RIP5, and RIP6 proteins (fused to nYFP). Bar = 50 µm.

**
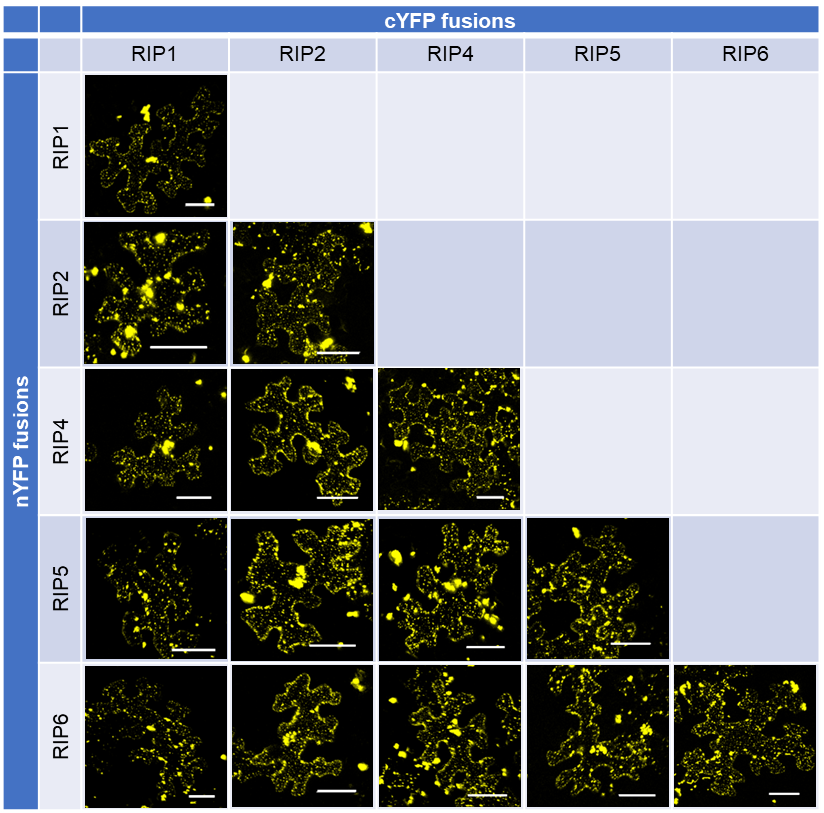
**

**Figure S2.** RIP proteins form homo- and hetero-dimers.

RIP1, RIP2, RIP4, RIP5, and RIP6 were each fused with cYFP and nYFP domains, and introduced into tobacco leaves in different combinations. Bar = 50 µm.


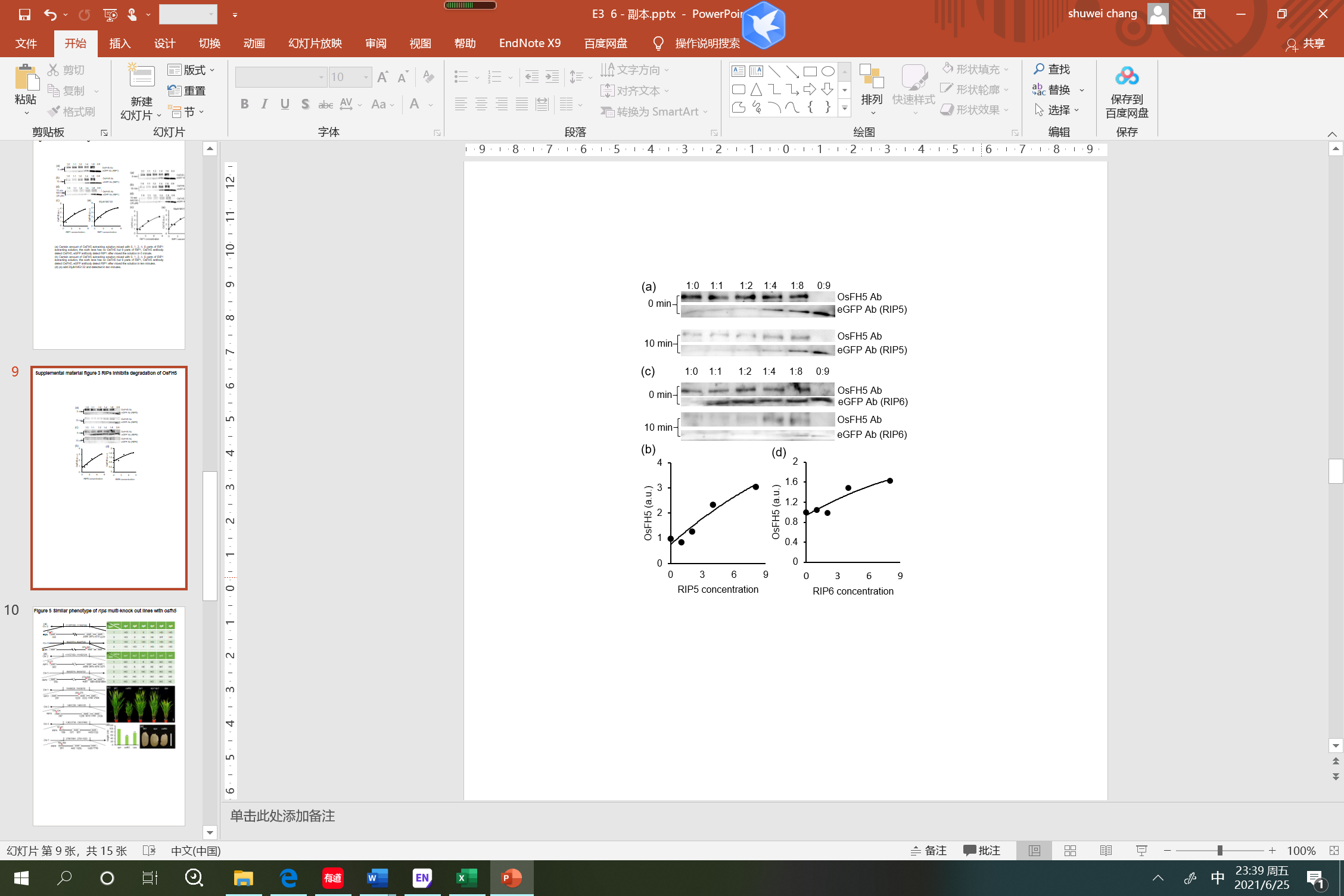


**Figure S3.** RIP5 and RIP6 inhibit degradation of RMD.

(a, b) RMD protein extract was mixed with different ratios (0, 1, 2, 4, or 8× vol) of (a) RIP5-eGFP extract or (b) RIP6-eGFP extract. The sixth lane contains a 9×vol of RIP1 extract with no RMD extract. Anti-RMD antibody (Ab) was used to detect RMD; anti-eGFP antibody was used to detect tagged RIP1. For (a) and (b), the upper panel shows protein amounts immediately after mixing (0 min); the lower panel, protein amounts 10 min after mixing.

(c, d) Quantification of western blot result from (a) and (b), respectively. a.u., arbitrary unit.


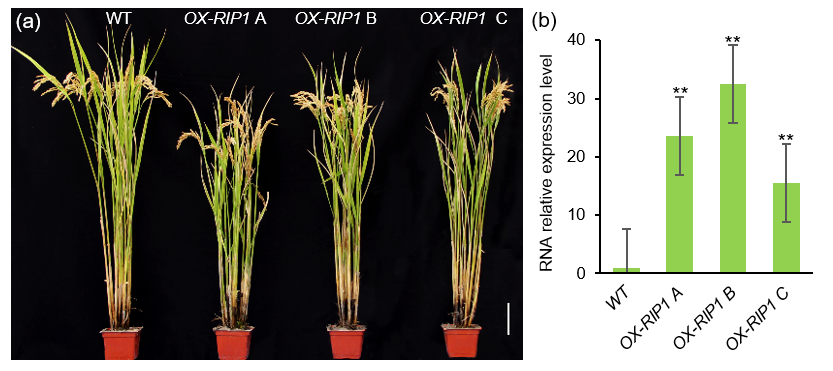


**Figure S4.** *RIP1* overexpression lines have no obvious phenotype.

(a) Phenotype of *RIP1* overexpression lines at heading stage compared with wild type (WT) 9522.

(b) *RIP1* expression in WT and overexpression lines. *P*< 0.01 (Student’s t-test).


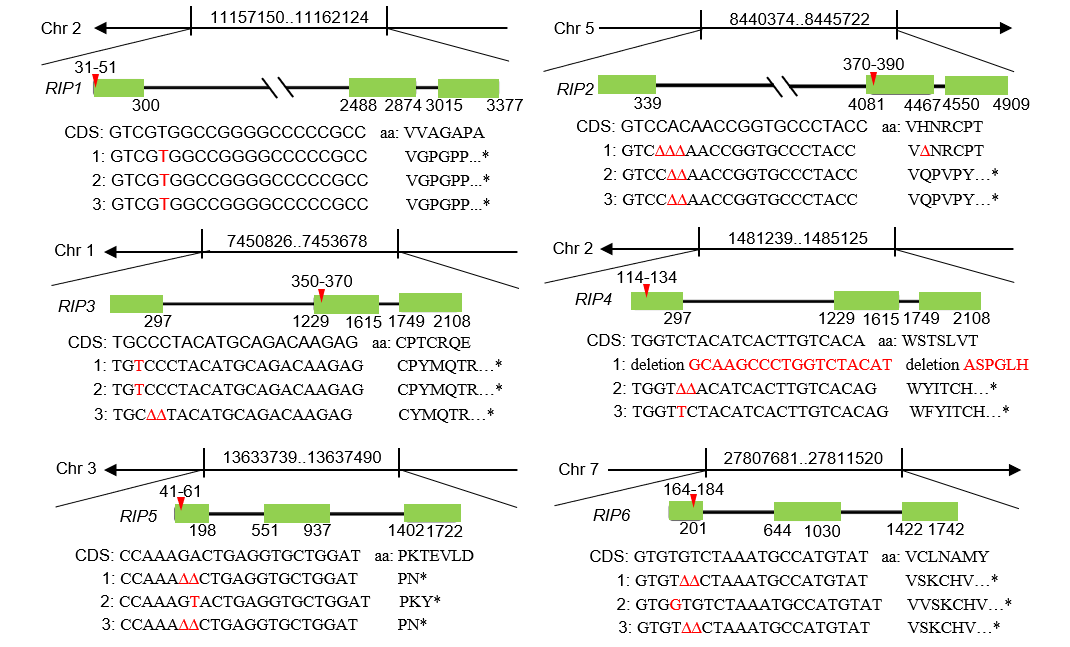


**Figure S5.** Changes in coding sequence (CDS) and protein amino acid (aa) sequence for 3 independent mutants for the 6 *rip* genes.

All 6 *RIP* genes contain three exons (green boxes), on 5 different chromosomes (Chr; position and direction indicated). Numbers above arrows indicate sequence position in the rice locus; numbers below exons indicate cDNA sequence position in gDNA; mutation sites (CRISPR) are marked with red triangles; numbers above the red triangles given position of CRISPR primers in cDNA. Red letters mean base added; ∆ means base or amino acid deletion; * means translation stop; …* means translation stops after several amino acids.


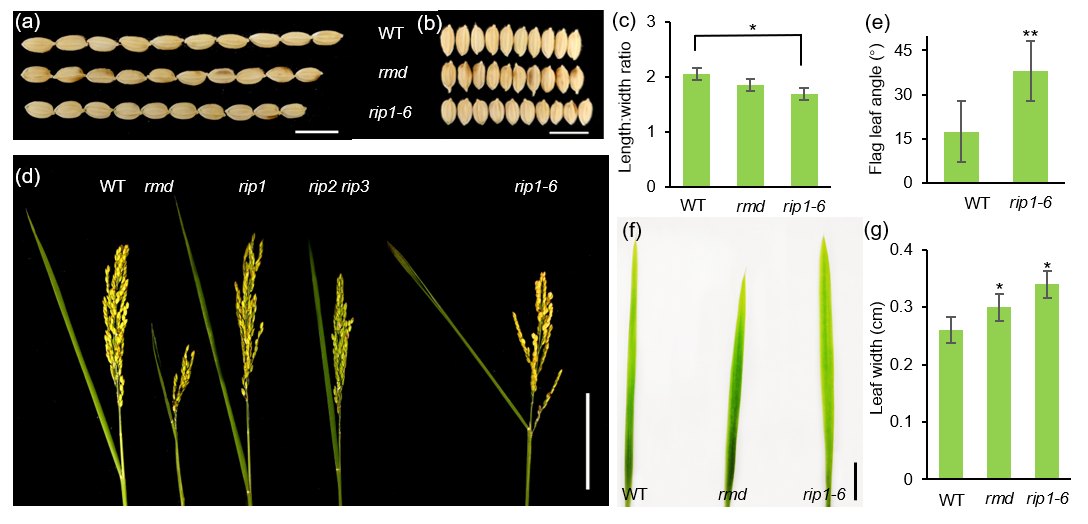


**Figure S6.** Other *rip1–6* phenotypes.

(a) Length and (b) width of wild type (WT), *rmd*, and *rip1–6* seeds. Bar = 1 cm.

(c) Length:width ratio of WT, *rmd*, and *rip1–6* seeds. n = 50, * *P*<0.05 (Student’s t-test).

(d) Phenotype of wider *rip1–6* flag leaves compared with WT, *rmd*, *rip1* and *rip1 rip2* flag leaves. Bar = 10 cm.

(e) The angle between flag leaf and stem increased in *rip1–6* compared with WT. n = 15, ** *P*<0.01 (Student’s t-test).

(f) Leaf width comparation among WT, *rmd* and *rip1-6*. Bar = 1 cm.

(g) Width of WT, *rmd*, and *rip1–6* flag leaves. n = 20, * *P*<0.05 (Student’s t-test).


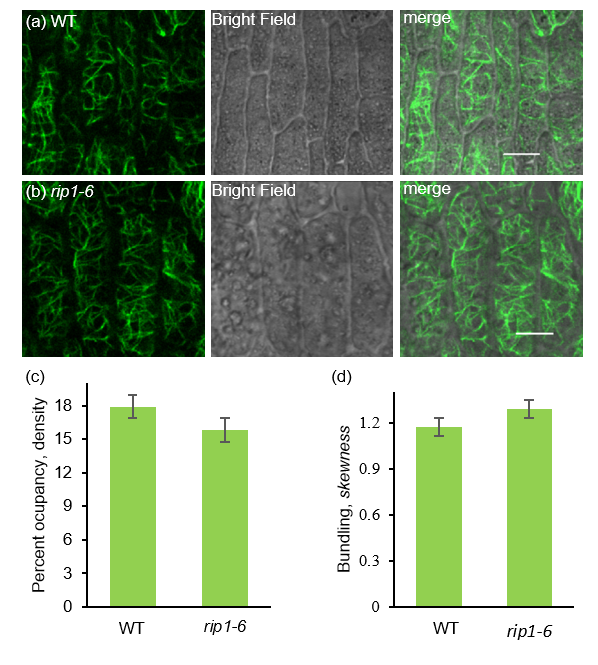


**Figure S7.** Coleoptile microfilaments of WT and *rip1-6* staining results

(a, b) WT and *rip1-6* coleoptiles (length 3-5mm) microfilaments stained with phalloidin. Bar = 10 µm.

(c) Actin filament abundance, or percentage of occupancy, in WT and *rip1-6* coleoptiles. n = 25.

(d) Actin filament bundling, or skewness, in WT and *rip1-6* coleoptiles. n = 25.
